# Perceived Individual and Systemic Impact of a Digital Wellbeing Package for Health and Care Workers Five Years Post-Release: A Qualitative Study

**DOI:** 10.3390/ijerph23040487

**Published:** 2026-04-13

**Authors:** Holly Blake, Neelam Mahmood, Ikra Mahmood

**Affiliations:** 1 School of Health Sciences, University of Nottingham, Nottingham NG7 2HA, UKikra.mahmood@nottshc.nhs.uk (I.M.); 2 NIHR Nottingham Biomedical Research Centre, Nottingham University Hospitals NHS Trust, Nottingham NG7 2UH, UK; 3 General Adult Psychiatry, Institute of Mental Health, Nottinghamshire Healthcare NHS Foundation Trust, Nottingham NG7 2TU, UK

**Keywords:** workforce, healthcare, digital technology, psychological wellbeing, leadership, organisational resilience, mental health support, qualitative research

## Abstract

**Highlights:**

**Public health relevance—How does this work relate to a public health issue?**

- Shows how digital mental health support may help buffer psychological strain among health and care workers during and after major public health emergencies.
- Addresses the ongoing challenge of workforce wellbeing within health systems operating under sustained pressure.

**Public health significance—Why is this work of significance to public health?**

- Provides qualitative evidence that this scalable, theory-informed digital intervention was perceived to produce lasting benefits for individual wellbeing, professional functioning, and organisational culture.
- Highlights participants’ accounts of how the digital resource informed leadership practices and supported system-level approaches to resilience.

**Public health implications—What are the key implications or messages for practitioners, policy makers and/or researchers in public health?**

- Suggests that digital wellbeing tools can be integrated into long-term workforce wellbeing strategies, complementing traditional support approaches.
- Indicates that interventions promoting collective and organisational responsibility for resilience may strengthen psychological safety and improve team culture across health systems.

**Abstract:**

This study explores health and care workers’ perceptions of the longer-term influence of a rapidly developed digital support package designed to promote psychological wellbeing during the COVID-19 pandemic. A qualitative design was used, involving semi-structured interviews with 20 health and care professionals, including frontline clinicians and senior leaders, who had used and disseminated a theory-informed digital wellbeing package, accessed globally by 82,425 users within its first year. Interviews conducted in 2025 examined participants’ accounts of perceived effects at individual, professional, and organisational levels. Data were analysed using reflexive thematic analysis informed by public mental health, organisational resilience, and implementation science perspectives. Four themes were identified: enhanced psychological wellbeing and coping; changes to professional practice and fatigue management; reframing resilience as a collective and organisational responsibility; and the sustainability and ongoing relevance of the resource beyond the pandemic. Participants described experiences such as reduced stress and anxiety, improved sleep and emotional regulation, sustained use of cognitive–behavioural strategies, and perceived improvements in functioning at work. Some participants also reported that the resource informed their thinking about leadership, psychological safety, and wellbeing practices, and described its continued relevance five years post-release. These qualitative findings illustrate how the digital wellbeing intervention was experienced by participants and how they interpreted its relevance over time. The study suggests that digitally delivered, theory-informed resources may have perceived value for individual capacity building, professional practice, and organisational approaches to resilience within health systems facing ongoing structural pressures.

## 1. Introduction

The COVID-19 pandemic placed unprecedented psychological, ethical, and operational pressures on health and care workers worldwide. Healthcare professionals experienced sustained high workloads, redeployment, staff shortages, moral distress, rapidly evolving clinical guidance, and repeated exposure to death and suffering [1,2,3,4,5]. Systematic reviews conducted during the pandemic reported elevated prevalence of anxiety, depression, insomnia, and burnout among frontline staff [6,7]. Moral injury—distress arising from perceived violations of deeply held ethical or professional values—also emerged as a significant concern [8].

Although workforce wellbeing challenges pre-dated COVID-19, the pandemic amplified longstanding structural pressures within health systems, including chronic understaffing, high emotional labour, and increasing service demand [9]. Prior to 2020, concerns about burnout, retention, and psychological safety were already prominent within United Kingdom (UK) workforce policy [10]. The pandemic exposed the fragility of traditional support mechanisms and highlighted the interdependence between staff wellbeing, patient safety, and system resilience. The “quadruple aim” framework (which extends health system performance metrics to include workforce wellbeing alongside patient experience, population health, and cost-effectiveness) provides an important conceptual lens for understanding these interdependencies [11].

### 1.1. Digital Wellbeing Interventions in Emergency Contexts

In response to escalating psychological distress, governments and health organisations rapidly introduced a range of wellbeing initiatives, including helplines, peer-support models, psychological first aid, and digital mental health interventions [12]. Digital resources were particularly attractive due to their rapid deployability, scalability, and low marginal cost [13,14]. In the UK, the NHS People Plan [15] and subsequent Staff Mental Health and Wellbeing Framework [16] emphasised the need for accessible, psychologically informed support, including digital provision. Internationally, the World Health Organization [17] identified workforce protection and wellbeing as central to health system resilience.

However, a scoping review conducted in 2020 [18] identified no high-quality, theory-based interventions for the well-being of healthcare workers during a pandemic or other crisis. The review searched the literature up to June 2020 (updated in October 2020) and identified only 13 relevant papers across all pandemics and crises; most were not digital nor designed for COVID-19; they related to earlier crises and were sparse overall. Another evidence review of interventions to support the mental health and well-being of front-line healthcare workers in hospitals during pandemics (with searches between 2000 and 2022) confirmed that early-pandemic digital support tools for healthcare workers were largely absent or unreported, with the development of most support tools emerging later [19,20]. In March 2020, there was an urgent need for rapid development in direct response to the absence of existing digital psychological wellbeing resources for healthcare workers when COVID-19 began [21].

Primarily due to the need for rapid response interventions during a crisis, evidence from early in the pandemic more commonly focused on short-term psychological outcomes, feasibility, or user satisfaction [21,22]. Less attention has been paid to how digital wellbeing interventions at this time influenced professional practice, leadership behaviours, organisational culture, and longer-term workforce sustainability.

This gap is significant because workforce wellbeing is not solely an individual-level phenomenon; it is embedded within complex organisational systems. Interventions that target coping skills alone risk reinforcing individualised models of resilience that obscure structural contributors to distress [19,23]. A conceptual advance is therefore needed to understand how digital wellbeing resources may function within broader organisational and policy ecosystems.

### 1.2. Conceptual Framework

This study is informed by three complementary conceptual perspectives: public mental health prevention models, organisational resilience theory, and implementation science.

(i) Public Mental Health and Capacity Building

Public mental health frameworks emphasise prevention, early intervention, and skills development at population scale [24]. Rather than focusing exclusively on treatment of diagnosable disorders, preventive approaches aim to strengthen coping, psychological literacy, and self-efficacy across workforces. Digital resources are particularly suited to this approach because they enable broad reach and standardised delivery. From this perspective, impact is conceptualised not only as symptom reduction but also as capacity building, including sustained cognitive reframing and behavioural change.

(ii) Organisational Resilience and Systems Thinking

Organisational resilience theory conceptualises resilience not as an individual trait but as a dynamic property of systems characterised by adaptability, learning, and supportive leadership [25]. Within healthcare, resilience involves both frontline coping and structural conditions that enable safe, sustainable practice [25,26]. Increasingly, UK regulatory and policy documents, including the NHS Long Term Workforce Plan [27] and the Care Quality Commission’s workforce wellbeing review [28], emphasise that workforce wellbeing is foundational to organisational performance and patient safety. By analysing digital wellbeing interventions through the lens of organisational resilience theory, we can evaluate how they may influence leadership culture, psychological safety, and shared responsibility for wellbeing, rather than viewing wellbeing solely as an individual-level issue.

(iii) Implementation Science and Normalisation

Implementation science provides a further lens for understanding how interventions become embedded within routine practice. Normalisation Process Theory [29] suggests that interventions are sustained when they make sense to users (coherence), are supported through engagement (cognitive participation), are operationalised in practice (collective action), and are appraised as valuable (reflexive monitoring). Emergency-deployed digital resources are rarely evaluated through this lens. Examining perceived long-term impact allows assessment of whether and how such interventions became normalised within professional routines, leadership practices, and organisational discourse.

### 1.3. Intervention Context

A digital support package [21,30] was developed rapidly in response to the COVID-19 pandemic and made freely available online. Designed to promote psychological wellbeing among health and care workers, the intervention drew on evidence from cognitive behavioural therapy (CBT), occupational wellbeing, stress and anxiety management, and sleep and fatigue science. It offered psychoeducational content, practical coping strategies, and guidance for managing stress, anxiety, fatigue, and emotional responses during crisis conditions.

**Figure 1:**
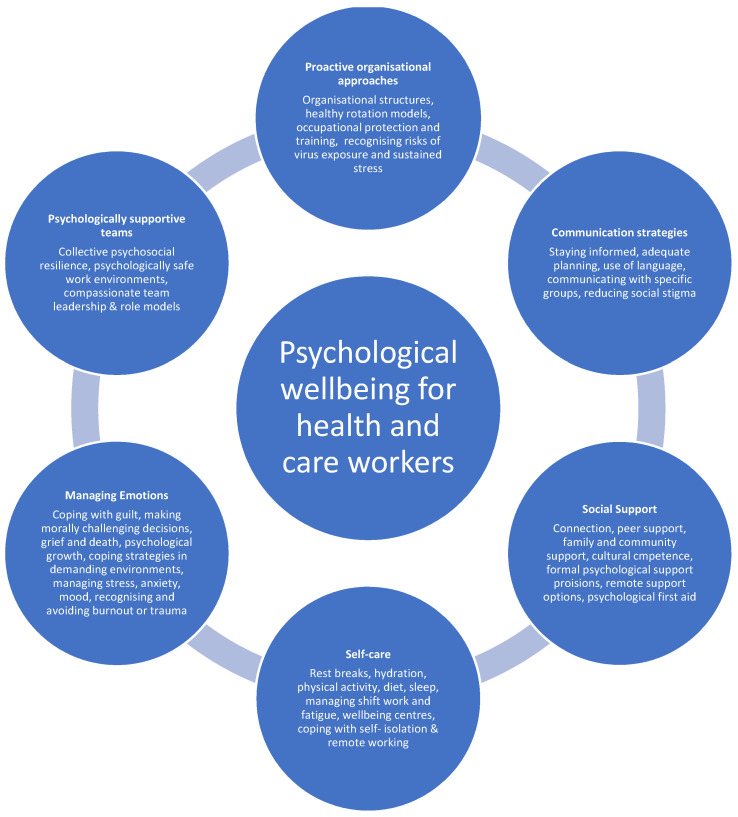
Content of the digital package for mitigating the impact of COVID-19 on health and care workers (reproduced with permission, from [31]).

Released within three weeks of the UK pandemic outbreak, the package included resources for both individual staff and team leaders. Content (Figure 1) covered creating psychologically safe work environments, effective communication, reducing social stigma, peer and family support, and signposting through psychological first aid (PFA). It also promoted self-care strategies such as rest, work breaks, managing fasting periods, sleep, shift work, fatigue, and healthy lifestyle behaviours, alongside guidance for managing emotions including moral injury, guilt, grief, fear, anxiety, depression, and burnout. Developed with input from mental-wellbeing experts and frontline practitioners, the package also directed users to public mental health guidance. Dissemination occurred through professional, organisational, and charitable networks, supporting broad and rapid uptake.

Early evaluations demonstrated that the digital package was appropriate, meaningful, and relevant to health and care workers and healthcare trainees, with high intervention fidelity, strong usability, and perceived utility [21,31]. Its rapid dissemination reflected the urgent demand for accessible psychological support during the early pandemic period: the resource was accessed 17,633 times in its first week and reached 82,425 users within 12 months (Figure S1). This widespread uptake occurred through professional, organisational, and charitable networks, which incorporated the resource into local and national workforce wellbeing offers.

The broader visibility of the intervention is also reflected in subsequent bibliometric and policy-level indicators. Two independent bibliometric analyses identified the original publication as the most globally cited paper in the fields of digital interventions, e-learning, and mental health during and after the COVID-19 pandemic [32,33]. In addition, the resource has been cited in 16 policy documents (as of March 2026) across multiple countries and intergovernmental organisations, suggesting that it contributed to wider discourse on workforce wellbeing during crisis conditions. Its global reach and communication-focused design was also recognised through a 2024 UK Hidden REF award for Communicative Outputs [34].

These indicators provide contextual information that helps explain the breadth of exposure among participants and the diverse organisational settings in which the resource was encountered. Given that the present study examines perceived long-term and system-level influence, it is important to situate participants’ accounts within the wider dissemination landscape that shaped how the intervention circulated and became embedded within practice. Despite this early evidence of reach, fidelity, and immediate relevance, the perceived longer-term individual and organisational impacts of the intervention had not previously been explored.

### 1.4. Study Aim and Contribution

The study addresses three interrelated questions:

1. How did the intervention influence psychological wellbeing and coping?
2. Did engagement affect professional practice and leadership behaviour?
3. Were impacts sustained and embedded within organisational contexts beyond the acute crisis phase?

By examining perceived impacts five years after release, this study extends beyond immediate crisis-response evaluation and contributes to the conceptual understanding of digital wellbeing interventions as tools for population-level capacity building, the potential of digital resources to influence organisational resilience and culture, and the mechanisms through which rapidly deployed interventions may become normalised within complex health systems. In doing so, the study responds directly to UK and international policy priorities concerning sustainable workforce wellbeing [24,27] and offers empirical insight into how digital interventions may generate both behavioural and systemic effects.

## 2. Materials and Methods

### 2.1. Study Design

A qualitative study design was employed using semi-structured interviews to explore participants’ experiences and perceptions of impact. The study adopted an interpretivist approach, recognising that impact is socially constructed and best understood through participants’ accounts. Reporting was guided by the Consolidated Criteria for Reporting Qualitative Research (COREQ: [35]) (Table S1).

### 2.2. Participants and Recruitment

Twenty participants were purposively recruited through professional networks for health and social care (including professional bodies, special interest groups, and organisational networks) that had received the digital package during the pandemic. Because this was also a convenience approach, it was not possible to determine how many individuals saw the invitation to participate. Eligibility criteria required participants to be health or care professionals, managers, or leaders who had accessed and disseminated the package, and the invitation explicitly welcomed both positive and critical views. Sampling was guided by the concept of information power [36], with adequacy judged by the focus of the study aim, the specificity of the sample, the depth and quality of interview dialogue, the interpretive orientation of reflexive thematic analysis, and the team’s qualitative expertise. Although one individual initially agreed but did not respond to scheduling, 20 participants ultimately completed interviews. Despite variation in roles and geographical location, interviews consistently generated rich, relevant accounts, and the team concluded that 20 interviews provided sufficient information power to support a robust and nuanced analysis. The final sample included doctors, nurses, allied health professionals, managers, and senior leaders, all of whom had accessed the package at its release and were re-sent the link during recruitment.

### 2.3. Data Collection

Semi-structured interviews were conducted by a single interviewer (IM) online via Microsoft Teams between 13 May and 30 July 2025. Interviews lasted between 14 and 111 min (average 38 min), no-one else was present during the interviews, and the interviewer took field notes during and immediately after the discussion. Of interviewees (10 men, 10 women), eight were service providers serving health and/or social care, 16 were clinicians (healthcare professionals from medicine, nursing and allied health disciplines) and six concurrently worked in health education roles. Fourteen interviewees held roles with local, regional or national influence, while six held roles with international influence (two of whom were based outside of the UK). Participant characteristics are shown in Table 1.

An interview guide (Text S1) explored participants’ experiences of using or sharing the package, perceived impacts on wellbeing and practice, and views on sustainability and relevance beyond the pandemic. The guide was developed by the project team and pilot tested with five healthcare professionals who were not involved in the study. Interviews were audio-recorded with consent and transcribed verbatim by one of two researchers (NM, IM), with transcripts checked by the interviewer for accuracy (IM). Participant feedback was sought with a small, randomly selected subset of approximately 10% of participants. This process was used not as a validation check, but to invite reflections that could enrich our interpretive engagement with the data and ensure that participants felt their experiences had been represented respectfully. Feedback was considered alongside, rather than as a test of, the developing analysis. Consent was taken prior to the interview, using an online form; no repeat interviews were undertaken.

### 2.4. Data Analysis

Data were analysed using reflexive thematic analysis, following Braun and Clarke’s six-phase approach [37,38]. Two researchers engaged collaboratively with the dataset, not to establish reliability through independent coding, but to deepen reflexive interpretation and bring diverse analytic sensibilities to the development of themes.

The analytic process was iterative and immersive. Researchers engaged in repeated readings of the transcripts to support deep familiarisation and to begin noticing early patterns of meaning. Coding was conducted inductively, with attention to features of the data that appeared conceptually or experientially significant.

As analysis progressed, these early insights were shaped into evolving patterns of meaning and, subsequently, into candidate themes. Theme development was refined through ongoing reflexive dialogue within the research team, which supported interpretive depth, critical examination of assumptions, and openness to multiple perspectives. The aim was not consensus or the production of a stable coding frame, but the generation of rich, nuanced themes grounded in the data. Throughout, researchers attended to how their disciplinary backgrounds, professional experiences, and positionalities influenced the analytic process.

To support transparency, we outline the thematic structure generated through this process. This representation is descriptive rather than hierarchical, reflecting the fluid, iterative, and meaning-centred nature of reflexive thematic analysis.

#### 2.4.1. Representation of Theme Development

The thematic structure reflects the breadth and nuance of participants’ experiences with the digital support package. Four overarching themes convey the central patterns of meaning constructed through the analysis: (1) Enhanced Psychological Wellbeing and Coping, (2) Changes to Professional Practice and Fatigue Management, (3) Reframing Resilience as a Collective and Organisational Responsibility, and (4) Sustainability and Ongoing Relevance Beyond the Pandemic. These themes represent interpretive insights rather than a hierarchical coding framework, and the analytic narrative highlights the complexity and diversity within participants’ accounts.

The first theme captures participants’ descriptions of strengthened emotional functioning and coping capacity, including experiences of validation, reduced stress, greater psychological stability, and the continued use of strategies such as normalisation, cognitive reframing, grounding, and enhanced self-awareness.

The second theme reflects shifts in professional behaviour and practice. Participants described changes in fatigue management, clinical performance, emotional regulation at work, supervisory approaches, and wider system-level adaptations. Their accounts highlighted new routines, altered ways of managing cognitive and emotional load, and organisational engagement with evidence-informed practices.

The third theme illustrates a move toward collective and organisational understandings of resilience. Participants emphasised psychologically informed leadership, the normalisation of vulnerability, and the embedding of wellbeing within organisational structures. These insights were shaped by evolving discourse, supervisory relationships, and workplace practices.

The final theme concerns the perceived durability and adaptability of the resource. Participants discussed its relevance to ongoing pressures, patterns of re-engagement, cultural and international applicability, digital sustainability, and cost-efficiency. They also reflected on structural pressures, changing working patterns, and the integration of wellbeing practices into professional identity and organisational culture.

Overall, the themes offer an interpretive map of the data, illustrating how participants’ accounts informed broader conceptual insights. This representation aligns with the flexible, iterative, and meaning-centred principles of reflexive thematic analysis.

#### 2.4.2. Reflexivity

All members of the research team were trained in good clinical practice, qualitative methods, and interview techniques. To minimise allegiance effects and confirmatory bias, two independent researchers, who were not involved in developing the digital package, conducted all interviews, completed the transcriptions, and contributed to the analysis and interpretation. The primary interviewer and coder was a female medical doctor working in public-sector health services; participants were informed of her professional background and interest in health research (credentials in Table S1). The second coder and transcriber was a female public-sector health researcher with a background in biomedical sciences and prior industry experience. Neither researcher had any prior relationship with participants. The lead author, a female health psychologist and experienced qualitative researcher, oversaw the study and supported the analytic process, and is the author of the digital package under discussion.

### 2.5. Ethical Considerations

Ethical approval was obtained from the University of Nottingham Faculty of Medicine and Health Sciences Research Ethics Committee (Ref: FMHS-118-0425, approved on 30 April 2025). Participants provided informed online consent and were assured of confidentiality and anonymity. Identifying details were removed from transcripts.

## 3. Results

**Figure 2:**
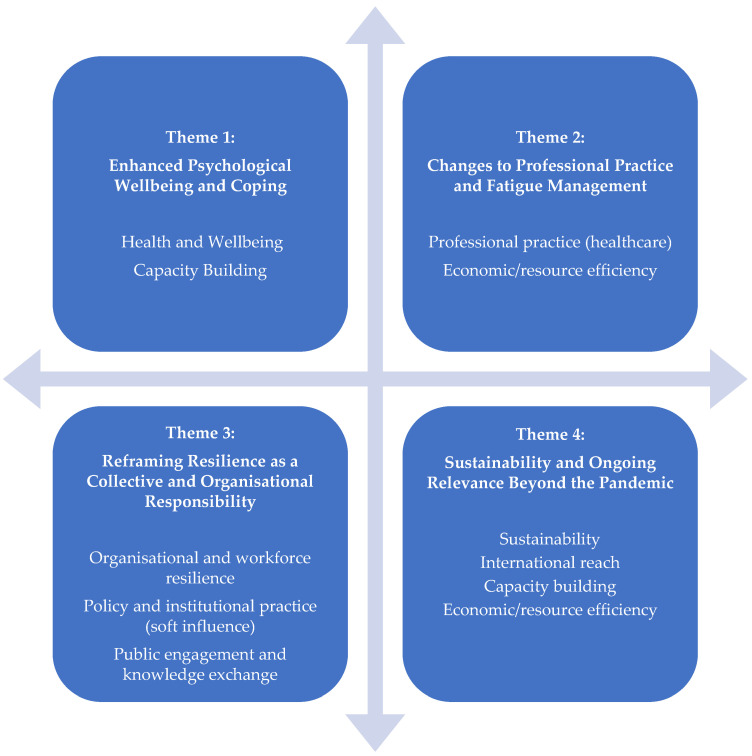
Themes mapped to impact domains.

**Figure 3:**
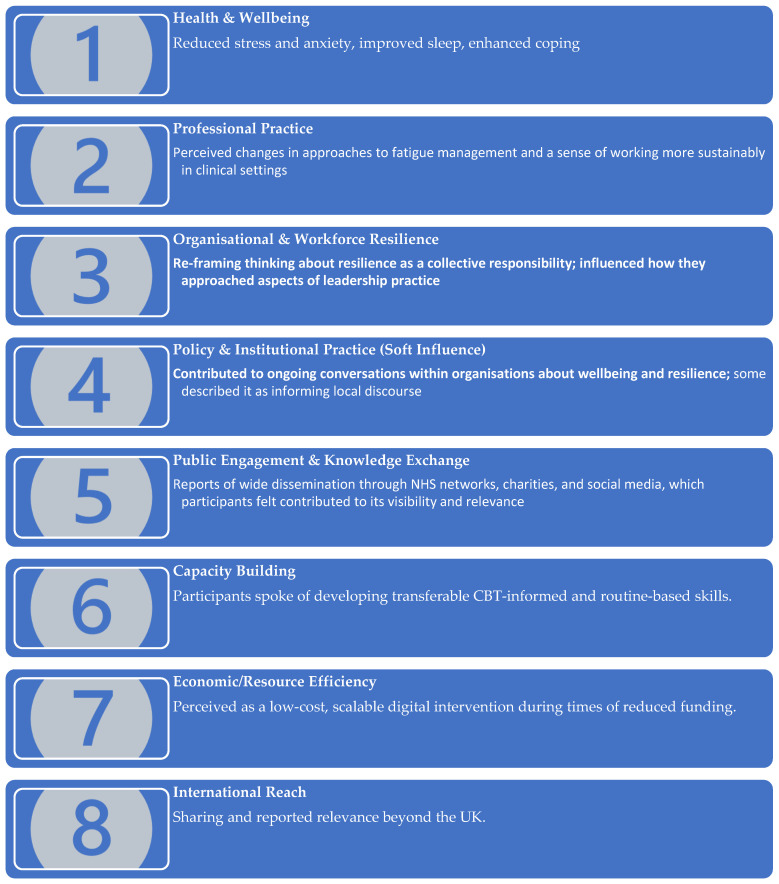
Eight impact domains mapped to areas of evidence from interviews.

Analysis identified four interrelated themes capturing participants’ interpretations of how the digital support package influenced them, including perceived effects on individual wellbeing, professional practice, organisational relationships, and broader understandings of resilience, capacity building, and sustainability. Figure 2 shows how themes map to impact domains. Figure 3 shows how those domains are evidenced in the data. 

### 3.1. Theme 1: Enhanced Psychological Wellbeing and Coping

Engagement with the digital support package was consistently perceived by participants as contributing to meaningful improvements in psychological wellbeing. Accounts described wide-ranging emotional benefits, including reduced distress, strengthened coping capacity, and a greater sense of psychological stability during an unprecedented period of uncertainty. These reported changes encompassed both immediate relief during the acute phases of the pandemic and more enduring shifts in attitudes and coping practices.

A prominent sub-pattern concerned the normalisation of emotional responses to crisis. Participants frequently described the content as reducing self-blame, challenging perceptions of personal inadequacy, and helping them reframe their emotional reactions as understandable in context. This validation appeared to reduce stigma surrounding psychological distress and enabled participants to approach their wellbeing with greater openness:

“It made me realise that feeling anxious didn’t mean I was failing. It was a normal response to an abnormal situation.”

(P105, Female)

“It validated what I was feeling, that this wasn’t me not coping, it was a really difficult situation.”

(P101, Female)

Alongside this emotional validation, participants described tangible reductions in stress and psychological intensity. Several reported feeling “less stressed”, “more energised”, and “thriving” (P103, Female) following engagement with the package. Others noticed clearer reductions in overwhelm, reflecting a shift toward greater psychological capacity and a sense of being better able to manage demands:

“It reduced my stress levels at the time.”

(P106, Female)

This sense of containment was often linked to the structure and reassurance provided by the materials, particularly during high-pressure periods. Participants described the content as offering cognitive and emotional grounding:

“It just made everything feel a bit more manageable, like there was a structure to fall back on.”

(P111, Male)

“It sort of steadied me. Things felt less chaotic in my head.”

(P108, Female)

For those working in environments of high mortality or ethical conflict, the targeted content on mindfulness, bereavement, and moral injury held particular significance. Participants described these modules as giving language and legitimacy to experiences they had struggled to communicate:

“What is mindfulness and how can I practice mindfulness? … it may decrease my stress, anxiety…”

(P107, Male)

“…trying to… switch off after and dealing with… a lot of death and dying.”

(P108, Female)

“I knew there was a module on grief and death… understanding those flashbacks… made more sense.”

(P113, Male)

“The bit about moral injury really resonated. It put words to something I hadn’t been able to articulate.”

(P109, Female)

Beyond emotional reassurance, participants emphasised the acquisition and active application of practical coping strategies. CBT-informed guidance, routines to support sleep and recovery, and structured techniques for cognitive reframing were frequently cited as particularly helpful:

“It helped me put routines in place, especially around sleep and switching off after shifts.”

(P107, Female)

“Trying to be more organised… staying active… reflect and debrief on difficult days…”

(P101, Female)

“I definitely challenge my thinking more now instead of spiralling.”

(P114, Female)

Participants also highlighted the transferability and longevity of these skills. Many reported that approaches first adopted during the pandemic were still in use five years later:

“It gave me tools I still use now, the CBT bits especially. I think differently about stress.”

(P114, Female)

“Those strategies didn’t stop when COVID eased, I still go back to them when work ramps up.”

(P112, Male)

While some participants already possessed strong self-care knowledge due to their professional roles, others described significant increases in self-awareness and psychological insight, particularly around stress recognition and early warning signs:

“Even when I didn’t keep up all the routines, I was more aware of when I was burning out.”

(P112, Male)

“It changed how I see my own wellbeing. I notice the warning signs earlier now.”

(P110, Male)

Across accounts, a shift toward self-compassion and more forgiving self-evaluation was notable:

“I’m kinder to myself than I was before.”

(P115, Male)

“I don’t feel guilty anymore for needing rest, I see it as part of doing my job safely.”

(P114, Female)

Participants also identified potential areas for improvement, such as adding images, diagrams, videos, or role-play scenarios, while emphasising the long-term value of the content:

“There’s loads of stuff… relevant in our day-to-day work… it could be applicable in any situation.”

(P102, Male)

“Maybe just some sort of videos… role plays or scenarios…”

(P101, Female)

A minority highlighted attitudinal barriers that limited uptake in some settings:

“I sent it to our management team… I suspect it was looked at and then disappeared…”

(P102, Male)

Overall, engagement with the digital support package was widely experienced as improving psychological wellbeing, offering emotional validation, reducing distress, and strengthening coping capacity during periods of acute uncertainty. Participants described feeling more grounded, less overwhelmed, and better able to regulate their emotions, while CBT informed strategies and routines supported longer term shifts in self-awareness, stress recognition, and self-compassion. Many continued to use these techniques years later, indicating long-lasting behavioural change. These individual level gains in coping and emotional regulation formed the basis for subsequent changes in professional practice, explored in Theme 2.

### 3.2. Theme 2: Changes to Professional Practice and Fatigue Management

Participants described how engagement with the digital support package prompted notable and sustained changes in their professional practice, particularly among those working in clinical environments. These shifts extended beyond personal wellbeing and were often framed as essential to delivering safer, more sustainable care within high-intensity settings such as Intensive Care Units (ICUs), Critical Care Units (CCUs), and Emergency Departments (EDs). Across accounts, participants highlighted how the package enhanced their understanding of fatigue physiology, rest planning, and recovery strategies, which they perceived as translating into concrete behavioural adaptations in their working lives.

A prominent sub-pattern centred on the development of more intentional, structured approaches to fatigue management, including organised pre- and post-shift routines around activity, food, light exposure, and winding-down strategies. Drawing on the package’s CBT-informed guidance, individuals described planning behaviours in advance, recognising early signs of cognitive and emotional overload, and applying practical techniques to support recovery. These routines were experienced as helping participants shift from reactive coping to more deliberate self-management:

“I’m able to get better sleep during the day and feel less fatigued after nights.”

(P107, Female)

“I started planning recovery days properly instead of just collapsing and hoping for the best.”

(P106, Female)

“I became much more deliberate about what I was doing before and after nights, thinking about food, light, winding down.”

(P103, Female)

“It made me realise I can’t just power through anymore. I have to actively manage fatigue.”

(P102, Male)

These changes were not described as minor lifestyle adjustments but as critical components of maintaining cognitive sharpness, emotional stability, and clinical safety. Participants repeatedly linked improved fatigue management to perceived enhancements in performance at work, noting that better-rested clinicians were better able to regulate their emotions, sustain concentration, and avoid errors:

“I’m less irritable when I’ve slept properly. I can hold things better emotionally.”

(P111, Male)

“When I’m less exhausted, I’m more patient, more focused… that has to be better for patients.”

(P110, Male)

“You’re sharper. You make fewer small mistakes when you’re not completely drained.”

(P114, Female)

Another important thread concerned the emotional labour inherent in clinical work during the pandemic. Several participants described gaining clearer strategies for managing psychological strain, particularly through learning to differentiate between aspects of their role that were within their control and those that were not. This reframing appeared to reduce emotional overload and mitigate burnout risk:

“It helped me separate what I could control from what I couldn’t, which made the emotional load feel lighter.”

(P105, Female)

“I notice sooner when I’m reaching my [emotional] limit on a shift.”

(P116, Male)

Crucially, the perceived impact of the package extended beyond individual behaviour change. Senior clinicians and managers described adapting their supervisory and team-support practices in response to the guidance, reflecting a shift toward more relational and preventative approaches to team wellbeing. These changes included normalising conversations about fatigue, sleep, and recovery:

“I feel more comfortable talking about mental health and wellbeing within my team.”

(P112, Male)

“I’ve spoken with colleagues about… bereavement and death… burnout… workloads… Being able to direct people to the package… was quite a nice way to wrap the conversation up.”

(P101, Female)

“I check in differently now. I’ll ask about sleep and recovery, not just workload.”

(P109, Female)

“One of my roles is kind of line managing junior staff… I have been a bit more mindful of… psychological well-being… giving people time off the shop floor… if they need it.”

(P102, Male)

For some, the package also served as an evidence-based tool for advocating systemic improvements. Participants described using the resource to legitimise discussions about rota design, protected recovery time, and broader organisational responsibilities for staff wellbeing:

“It gave me something evidence-based to point to when we were discussing rota changes.”

(P108, Female)

“We are changing shift patterns… partly… trying to not break everyone… this has definitely fed into that kind of discussion.”

(P102, Male)

“Having that framework made it easier to justify protected recovery time after intense runs of shifts.”

(P115, Male)

As these individual and supervisory shifts accumulated, participants described broader ripple effects within teams and services. Learning from the package appeared to subtly reshape shared assumptions about what constitutes safe, sustainable clinical practice. Concepts such as proactive fatigue planning, recognising cognitive limits, and viewing recovery as integral to patient safety became reference points in discussions about staffing, workload, and professional expectations. This diffusion of new norms created conditions in which teams were more willing to question entrenched practices and foreground wellbeing in operational decisions.

The perceived value of the package was reinforced by widespread dissemination within teams, with participants sharing the resource proactively due to its practicality and relevance:

“We shared it across the team because it was something practical, not just a poster about resilience.”

(P113, Female)

Participants also emphasised the value of the package’s digital, low-cost, and scalable format, particularly in a context of shrinking wellbeing budgets and limited protected time for training:

“A consolidated resource… everything in one place.”

(P101, Female)

“Scalable without needing extra budget or external facilitators.”

(P104, Female)

“It’s sustainable because it doesn’t require time off for courses or funding for trainers.”

(P118, Female)

Overall, building on enhanced personal coping, participants reported substantial and sustained changes in their clinical practice, particularly around fatigue management, recovery planning, and emotional regulation at work. The package prompted more intentional pre- and post-shift routines, greater recognition of cognitive and emotional limits, and clearer strategies for managing psychological strain. These adaptations were perceived to improve concentration, emotional stability, and patient safety. The resource also influenced supervisory behaviours, normalising conversations about wellbeing, sleep, burnout, and recovery within teams. As these practices diffused, they contributed to broader shifts in how safe and sustainable clinical work was conceptualised, leading into Theme 3’s focus on organisational responsibility.

### 3.3. Theme 3: Reframing Resilience as a Collective and Organisational Responsibility

A central theme concerned a marked shift in how resilience was conceptualised within healthcare organisations. Participants, particularly those in leadership and managerial roles, described moving away from individualistic narratives of coping and toward a more collective, structural, and organisational framing of wellbeing. The digital package was frequently identified as prompting reflection on the systemic conditions required to sustain staff wellbeing and the responsibilities of leaders in shaping those conditions.

Many leaders reported that the intervention encouraged them to challenge long-standing assumptions that resilience is solely an individual trait. Instead, they described recognising resilience as something that must be actively supported through team processes, organisational practices, and wider cultural norms:

“It helped reframe resilience as something that needs to be supported by teams and organisations, not just individuals.”

(P104, Female)

“It made me think about what we as leaders are responsible for—not just telling people to look after themselves.”

(P112, Male)

“It shifted the narrative from ‘coping’ to ‘what are we putting in place to support people?’”

(P101, Female)

Participants contrasted this reframing with pre-pandemic discourses that emphasised endurance, stoicism, or mental toughness. Several reflected that the package legitimised a more compassionate understanding of resilience, helping them move away from narratives that implicitly blamed individuals for struggling under extreme conditions:

“We talk less about ‘being tougher’ and more about what the system needs to change.”

(P115, Male)

A related shift involved leaders modelling vulnerability and normalising open dialogue about psychological strain. Participants described becoming more willing to acknowledge difficulty, creating a more permissive environment for others to do the same, and making wellbeing a legitimate agenda item within team settings:

“I’m more open about saying when things are difficult. That gives other people permission.”

(P109, Female)

“It made wellbeing something we could actually discuss in meetings, not just something implied.”

(P113, Female)

Leadership practices also appeared to change at a relational level. Participants described adopting more intentional, psychologically informed approaches to supervision and communication, with a stronger focus on emotional check-ins and recognition of burnout warning signs:

“I check in differently now. I ask how people are really doing, not just whether the work is done.”

(P116, Male)

“It encouraged me to think about psychological safety as part of my job, not an optional extra.”

(P108, Female)

For some, these shifts extended into structural practices. The language and principles from the package were integrated into supervision, appraisals, and debriefing processes, with leaders reporting that the resource offered actionable guidance for embedding wellbeing into everyday organisational routines:

“I’ve used some of the principles in appraisals and team reflections.”

(P106, Female)

“It informed how we structured debriefs after particularly difficult shifts.”

(P103, Female)

The influence of the package also extended beyond individual departments. Participants described deliberate strategic dissemination across organisations, professional networks, and international settings. Dissemination occurred through both formal internal channels and informal professional networks:

“We circulated it through our directorate, and it went out in the staff bulletin.”

(P110, Male)

“It went to around 18,000 staff across the organisation once it was shared centrally.”

(P114, Female)

“I sent it to colleagues in other [hospital] trusts and to contacts in voluntary organisations.”

(P105, Female)

“I know people shared it as far as New Zealand and it was shared amongst their networks there as well.”

(P109, Female)

Social media was also highlighted as a key mechanism for diffusion:

“It was picked up on Twitter [now X] and that’s how it spread beyond our immediate circles.”

(P102, Male)

Although participants acknowledged the difficulty of quantifying downstream organisational outcomes during a global crisis, they consistently described the package as influencing discourse, validating collective experience, and establishing a shared, non-judgemental language around resilience:

“It acknowledged what people were going through collectively. That felt powerful.”

(P118, Female)

“It changed the tone of conversations. Wellbeing became something legitimate to talk about.”

(P113, Female)

“It gave us a shared language around resilience that wasn’t blaming.”

(P111, Male)

Credibility was also central to its perceived impact. Participants highlighted that the evidence-based nature of the resource helped it gain traction and trust within organisational environments:

“Because it was research-based, it carried weight. It wasn’t just another wellbeing email.”

(P107, Female)

Overall, participants, particularly those in leadership roles, described a shift away from individualistic narratives of resilience toward a more collective, systemic framing of wellbeing. The package encouraged leaders to recognise organisational responsibility for creating conditions that support staff, legitimising open discussion of psychological strain and embedding wellbeing principles into supervision, appraisals, and team routines. Dissemination across departments and international networks further reinforced a shared, non-judgemental language around resilience. This cultural reframing provided the foundation for understanding the package’s continued relevance beyond the immediate crisis, as explored in Theme 4.

### 3.4. Theme 4: Sustainability and Ongoing Relevance Beyond the Pandemic

A consistent thread across interviews was the view that the digital support package retained relevance long after the acute crisis of COVID-19 had passed. Although originally created as an emergency response, participants described the content as mapping onto enduring challenges within the health and social care landscape. Rather than perceiving the package as temporally bound to a specific moment in the pandemic, participants framed it as a resource aligned with structural, ongoing pressures that continue to shape workforce wellbeing.

Participants emphasised that many of the psychological demands addressed in the package—stress, anxiety, uncertainty, and cumulative fatigue—remained integral to their everyday working lives:

“The principles are universal—stress and anxiety didn’t disappear when the pandemic ended.”

(P101, Female)

This sense of continuity was echoed widely, with many describing the resource as relevant to the post-pandemic reality of persistent workforce strain, capacity shortages, and sustained operational pressure:

“This [package content] isn’t just about COVID. It’s about how we work in healthcare now.”

(P116, Male)

Several participants linked this enduring relevance to the intensification of system pressures rather than their resolution. They highlighted ongoing backlog management, depleted staffing, and chronic burnout risk as factors that kept the resource highly applicable:

“The pressure hasn’t gone away. If anything, it feels more relentless now.”

(P111, Male)

“We’re still dealing with backlog, staffing gaps, and burnout… the content still fits.”

(P106, Female)

Participants in leadership and education roles further identified contemporary challenges related to hybrid working models, dispersed teams, and new forms of professional isolation. They felt the resource supported staff navigating these evolving structures:

“It’s relevant in a hybrid world, people are isolated in different ways now.”

(P112, Male)

“Even outside of clinical redeployment, there’s still anxiety and uncertainty in teams.”

(P103, Female)

Significantly, participants described returning to the resource at different stages of their careers or during subsequent periods of heightened pressure, demonstrating perceived long-term utility and reusability:

“I’ve gone back to it at different stages when things have ramped up again.”

(P114, Female)

“It wasn’t a one-off thing during lockdown; I still refer people to it.”

(P109, Female)

Sustainability was also understood in terms of the package’s flexibility and potential adaptability. Participants spoke about opportunities for translation, cultural contextualisation, and technological enhancement to maintain relevance across future contexts and diverse settings:

“It could easily be translated and used internationally.”

(P105, Female)

“The framework isn’t culturally specific; it would travel well.”

(P110, Male)

“An app version would make it even more accessible.”

(P107, Female)

“It could evolve with the times… the principles stay the same even if the context changes.”

(P115, Male)

Economic sustainability was another recurring theme. In a context of constrained wellbeing funding, participants highlighted the value of a low-cost, digital, non-facilitated resource that remained accessible without requiring recurrent investment:

“It’s sustainable because it doesn’t require new money every year, it’s just there, accessible.”

(P118, Female)

“There’s no facilitator needed, no study leave required, people can access it when they need it.”

(P102, Male)

“Given how stretched budgets are now, something like this is realistic.”

(P104, Female)

This contrasted strongly with more resource-intensive wellbeing interventions:

“We can’t keep funding workshops every year, but this is something that stays.”

(P113, Female)

The package’s ongoing relevance also extended beyond national borders. Participants described deliberate sharing across professional networks internationally, contributing to global dissemination and reinforcing the universal relevance of the content:

“It didn’t just stay local, colleagues abroad picked it up and shared it.”

(P108, Female)

“I know it reached people internationally through professional contacts.”

(P109, Female)

For several participants, sustainability was understood not only in terms of content longevity or cost efficiency but also in terms of cultural endurance. The package was perceived as contributing to an ongoing shift in organisational discourse about wellbeing; one that had persisted even after the acute crisis moment:

“It planted a seed. The conversation about wellbeing hasn’t gone backwards.”

(P116, Male)

“It normalised something that we’re still building on now.”

(P101, Female)

Overall, although developed as an emergency response, the package was viewed as highly relevant to ongoing workforce pressures, including work-related stress, cumulative fatigue, staffing shortages, and sustained operational strain. Participants continued to revisit the resource during later periods of heightened demand, and leaders highlighted its adaptability to changing work contexts including hybrid working, dispersed teams, and international contexts. Its low-cost, scalable, and non-facilitated design was seen as particularly valuable in settings with constrained wellbeing budgets. These features, combined with its contribution to longer-term cultural shifts around wellbeing, underscored its perceived sustainability and enduring utility within the post-pandemic health and care landscape.

## 4. Discussion

This study provides rich qualitative evidence of the perceived multi-level impact of a digital wellbeing support package developed for health and care workers during the COVID-19 pandemic [21,30]. Participants described improvements in psychological wellbeing, changes in professional practice, organisational reframing of resilience, and sustained relevance beyond the acute pandemic phase. Crucially, findings demonstrate both significance (depth of change) and reach (national and international dissemination). Figure S2 shows that across themes, the data demonstrate perceived depth of impact, breadth of reach, longevity, and economic efficiency.

### 4.1. Psychological Wellbeing and Alignment with Policy Priorities

Participants reported improvements in psychological wellbeing, including reduced stress and anxiety, enhanced emotional regulation, and increased coping self-efficacy. These findings are consistent with research documenting elevated rates of distress, burnout, and moral injury among healthcare professionals during the pandemic [1,2,3,4,5,6,7,8,39]. The sustained use of CBT-informed strategies and psychoeducation for many participants five years post-release suggests that this intervention was viewed by participants to result in durable cognitive and behavioural change.

This aligns not only with UK policy priorities but also with global calls to strengthen mental health support for health and care workers. International bodies such as the World Health Organization and the International Labour Organization [40,41] have emphasised the need for accessible, evidence-based psychological resources, proactive prevention, and organisational cultures that promote psychological safety. The UK government’s recognition of these priorities is reflected in the Health and Social Care Committee’s [42] inquiry into workforce burnout and resilience, which identified staff wellbeing as central to quality and sustainability in NHS services. Participants’ accounts of reduced stigma and normalised emotional responses resonate with the Committee’s call for psychologically safe and supportive cultures in healthcare settings.

Furthermore, the NHS People Plan [10,15] and subsequent Staff Mental Health and Wellbeing Framework [16] explicitly foreground proactive interventions to support mental health and psychological safety. These policies emphasise early, accessible support rather than after-the-fact remediation, a principle reflected in participants’ descriptions of the digital support package as a preventive and empowering resource. The Care Quality Commission’s (CQC) Workforce Wellbeing Insight Review [28] further acknowledges that staff wellbeing influences the quality of patient care and organisational performance. Participants’ reports of better emotional regulation and focused functioning at work because of using the package accentuate this nexus between employee wellbeing and service delivery.

### 4.2. Professional Practice, Patient Safety, and Regulatory Context

A key contribution of this study is evidence of the impact of this digital package on professional practice, particularly fatigue management, shift planning, and emotional self-regulation in high-intensity clinical environments. Participants linked improved sleep and fatigue management with safer clinical performance, a finding with direct implications for healthcare quality and safety.

This resonates with CQC’s regulatory emphasis that organisations must effectively manage staff workload, fatigue, and wellbeing to maintain safe services [28]. In the UK, NHS regulatory frameworks increasingly link staff wellbeing metrics with inspections and quality ratings, underlining the practical relevance of interventions that demonstrably improve workforce functioning. Internationally, the World Health Organization’s Global Strategy on Human Resources for Health [43] recognises health workforce wellbeing as integral to resilient health systems. The present findings extend this perspective by illustrating how digital interventions can support workforce sustainability and mitigate systemic strain.

### 4.3. Organisational Reframing of Resilience and Leadership Practice

A central theme surrounding the impact of this digital package was the reframing of resilience from an individual attribute to a collective and organisational responsibility. Participants described shifts in leadership discourse, supervisory practice, and team communication, moving away from messages of individual toughness toward systemic support and shared accountability.

This transition aligns with critiques of traditional resilience paradigms that risk individualising systemic problems [23,25]. It also reflects recommendations in UK policy documents such as the NHS People Plan [10,15] and the NHS Long Term Workforce Plan [27], which emphasise whole-system approaches to wellbeing, psychological safety, and inclusive leadership. Participants’ descriptions of embedding wellbeing conversations into team routines mirror the CQC’s emphasis on positive workplace cultures where staff feel “safe, supported, and valued” [28]. Such cultural shifts are now considered central to high-quality care and are increasingly measured as part of regulatory assessments.

Importantly, these developments also resonate with global policy priorities, including the World Health Organization and International Labour Organization’s [40] joint call for organisational-level action to protect mental health at work, promote supportive leadership, and embed psychosocial risk management as a core responsibility of employers. These international frameworks similarly emphasise that resilience should not be positioned as an individual burden but as a shared organisational and system-level commitment.

### 4.4. Sustainability, Scalability, and Systemic Relevance

Participants consistently emphasised that the package remained relevant in the post-pandemic context of chronic pressures, workforce shortages, hybrid working, and sustained high stress. Importantly, the package was described as both scalable and low-cost, requiring minimal ongoing organisational investment. These characteristics align with broader policy priorities. The NHS Workforce Plan [27] highlights scalable digital tools as essential in expanding support without proportionate increases in cost or workforce burden. Similarly, the UK Department of Health and Social Care [44] report on digital health highlights the potential of digital resources to enhance workforce wellbeing and capacity. International sharing through professional networks and social media indicates that the package has had reach far beyond the UK context. This international uptake aligns with WHO frameworks prioritising cross-national exchange of effective workforce wellbeing practices [45].

### 4.5. Strengths and Limitations

A key strength of this study is its deliberately diverse sample of 20 participants, spanning frontline clinicians, service providers, educators, managers, and organisational leaders across the UK and internationally. This breadth enabled exploration of how the digital package was experienced and interpreted across multiple layers of the health and care system, offering insight into individual, professional, and organisational domains of perceived impact. The five-year retrospective timeframe is an additional strength, allowing examination of sustained relevance and longer-term sense-making rather than short-term novelty effects. The qualitative, interpretivist design was well suited to these aims, generating rich, nuanced accounts of how the resource was used, valued, and embedded within participants’ professional contexts.

Several limitations must also be acknowledged. The study does not assess measurable changes in clinical performance, organisational indicators, patient safety, or staff wellbeing; such outcomes would require complementary quantitative or mixed-methods approaches. Recruitment through professional networks that had previously disseminated the digital package means the denominator of individuals who saw the invitation is unknown, and the sample is likely enriched for users who were positively engaged with the resource and for respondents in leadership-oriented roles. As a result, perspectives from non-users, disengaged users, or those with negative experiences are absent. These sampling characteristics, combined with the retrospective nature of the interviews, may introduce self-selection, recall, and social desirability biases. The findings therefore reflect participants’ perceptions of influence rather than evidence of long-term effectiveness or verifiable organisational change.

The five-year interval between release and interviews also raises the possibility that some reported changes may reflect broader cultural shifts in healthcare, evolving policy landscapes, or exposure to other wellbeing initiatives over time. Finally, because one author developed the digital package, there is potential for author allegiance to have shaped aspects of interpretation. This was mitigated through the use of independent interviewers and coders who were not involved in the package’s development, and through a reflexive analytic approach that foregrounded critical dialogue, positionality, and interpretive transparency.

Despite these limitations, the study provides valuable insight into how a rapidly deployed digital wellbeing resource was experienced by a diverse group of health and care professionals and leaders, and how they perceived its influence on their wellbeing, practice, and organisational contexts over a five-year period. The findings offer an important contribution to understanding the potential longer-term role of digital interventions within workforce wellbeing strategies and organisational resilience efforts.

### 4.6. Theoretical Contribution

This study contributes to conceptual understanding of digital wellbeing interventions within complex health systems in three key ways based on how participants interpreted and described the longer-term relevance of the resource. First, participants’ accounts extend public mental health prevention models by illustrating how a digitally delivered, theory-informed resource was experienced as supporting workforce-level capacity building, with some describing sustained cognitive and behavioural shifts beyond short-term wellbeing management. Second, when viewed through organisational resilience theory, participants’ narratives suggest that digital interventions may be understood as influencing not only individual coping but also thinking about cultural and leadership practices—particularly the reframing of resilience from an individual attribute toward a more collective, organisationally embedded responsibility. Third, from an implementation science perspective, participants’ descriptions of continued relevance, repeated engagement, and ongoing dissemination align with processes associated with normalisation within professional routines and organisational discourse.

Overall, these theoretically informed interpretations highlight how participants made sense of the role of this resource over time. Rather than evidencing system-level transformation, the findings show how a digital wellbeing intervention was perceived to resonate with, and potentially contribute to, existing frameworks concerned with workforce strategy, organisational culture, and health system resilience.

### 4.7. Implications for Practice and Future Research

The findings highlight the potential value of digital wellbeing resources, but their applicability beyond the study context will depend on wider structural and cultural conditions. Implementation research consistently shows that digital interventions succeed only when they align with local context, organisational readiness, and existing workflows [46,47]. National workforce policies, digital infrastructure, and professional norms vary considerably across health systems, and these factors—along with well-recognised implementation challenges such as variable digital literacy, competing organisational priorities, and uneven access to technology—are likely to shape both uptake and impact [48,49,50,51,52].

From an implementation perspective, organisations considering similar approaches may need to adapt content, delivery formats, and implementation strategies to ensure fit with local priorities, technological capacity, and workforce cultures. Implementation frameworks emphasise the importance of adaptability, co-design, and iterative refinement to enhance acceptability and sustainment [53,54]. Practical opportunities include developing app-based or mobile-first versions, updating materials to reflect evolving evidence and policy landscapes, and tailoring language and examples for different cultural or professional groups. Embedding such resources prospectively within broader organisational wellbeing strategies—rather than relying on ad hoc dissemination—may also strengthen their reach, fidelity, and sustainability [48,49,53].

Future research could examine how adapted versions perform in different health systems and use mixed-methods or longitudinal designs to test hypothesised links between digital wellbeing interventions and outcomes such as patient safety, staff retention, organisational climate, or workforce performance. Implementation studies could also explore mechanisms of action, including how digital support packages interact with contextual factors such as leadership behaviours, team climate, and organisational digital maturity. Such approaches would help clarify the pathways through which digital interventions exert influence and identify the contextual conditions that enable them to have meaningful, system-level impact [46,50,51,52].

## 5. Conclusions

This study provides qualitative evidence that a theory-informed digital wellbeing support package [18,27], developed during the COVID-19 pandemic, was experienced by participants as contributing to meaningful and, for many, enduring benefits across individual, professional, organisational, and international contexts. Participants described enhanced psychological wellbeing, shifts in professional behaviour, and perceived cultural change in how resilience and wellbeing are understood and enacted within organisations. The scalability, low cost, and continued relevance of this resource, amid ongoing workforce pressures five years later, suggest its perceived potential to support longer-term wellbeing initiatives, extending its usefulness beyond the pandemic response.

The findings indicate that this digital wellbeing tool, when grounded in behavioural science and designed for broad accessibility, was seen by participants to play a meaningful role within long-term workforce sustainability efforts. They also highlight the importance of embedding such interventions within UK workforce policy and regulatory agendas [15,16,24,25,36], while aligning with international priorities that encourage cross-national exchange of effective wellbeing practices [17,37]. Insights from this study support the positioning of this digital wellbeing intervention as a valued component, based on participants’ accounts, of modern workforce strategies, with the perceived potential to strengthen individual and organisational resilience, enhance professional functioning and contribute to healthier organisational cultures.

## Figures and Tables

**Table 1:** Participant characteristics.

ID	Gender	Role	Main Discipline(s)	Leadership	National/International	Interview Length
101	F	C	Medicine	N	N	21:45
102	M	C	Medicine	Y	N	23:58
103	M	C	Medicine	Y	N	15:03
104	M	SP	Psychology	Y	N	24:51
105	F	C/SP	Nursing	Y	N	14:36
106	F	SP	Public Health	Y	N	17:57
107	M	C/HE	Nursing	N	I	48:37
108	F	C	Nursing	Y	N	53:48
109	F	C/HE	Nursing	Y	N	108:32
110	M	C	Nursing	Y	N/I	49:01
111	M	C/HE	Nursing	Y	N	111:43
112	F	C/HE	Physiotherapy	Y	I	39:46
113	M	C	Medicine	N	N	30:19
114	M	SP	Management	Y	N	25:14
115	F	C/HE	Medicine	Y	N/I	30:57
116	M	SP	Management	Y	N	31:38
117	F	C/SP	Nursing	Y	N/I	38:03
118	F	C/SP	Nursing	Y	N/I	29:23
119	F	C/HE	Psychology	N	N	25:19
120	M	C/SP	Nursing	Y	N/I	21:49

Role: Clinician: C, Service Provider: SP, Health Educationalist: HE.

## Data Availability

Due to the sensitive nature of the data and the risk of participant identification, the datasets generated and analysed during the current study are not publicly available but may be accessed on reasonable request from the corresponding author.

## Supplementary Materials

The following supporting information can be downloaded at: https://www.mdpi.com/article/10.3390/ijerph23040487/s1, Figure S1: Global access data after 12 months; Figure S2: Integrated evidence of perceived research impact. Table S1: Consolidated criteria for reporting qualitative studies (COREQ): 32-item checklist; Text S1: Interview topic guide.

## Abbreviations

The following abbreviations are used in this manuscript:

COVID-19	Coronavirus pandemic
COREQ	Consolidated Criteria for Reporting Qualitative Research
CQC	Care Quality Commission
NHS	National Health Service
UK	United Kingdom
WHO	World Health Organization
