# Supplementary material for: Perceived Individual and Systemic Impact of a Digital Wellbeing Package for Health and Care Workers Five Years Post-Release: A Qualitative Study
Source: Int J Environ Res Public Health. 2026 Apr 13;23(4):487. doi: 10.3390/ijerph23040487 (PMC13116477; doi:10.3390/ijerph23040487)
Supplement: Supplementary file 1 [file ijerph-23-00487-s001.zip › Figure S1.pdf]

Figure S1: Global access data after 12 months

Project Details

Name: Psychological Wellbeing for Healthcare Staff
ID: 22794
Created on: 2020-03-31 00:00:00
Last modified on: 2020-07-06 00:00:00
Template: Xerte Online Toolkit
Player: HTML5

URL: https://xerte.nottingham.ac.uk/play_22794

Embed Code:

<iframe src="https://xerte.nottingham.ac.uk/play_22794" width="802" height="602" frameborder="0" style="position:relative; top:0px; left:0px; z-index:0;"></iframe>

Project size: 18.42 MB
Access: Public
Views: 82425
