# Supplementary material for: Perceived Individual and Systemic Impact of a Digital Wellbeing Package for Health and Care Workers Five Years Post-Release: A Qualitative Study
Source: Int J Environ Res Public Health. 2026 Apr 13;23(4):487. doi: 10.3390/ijerph23040487 (PMC13116477; doi:10.3390/ijerph23040487)
Supplement: Supplementary file 1 [file ijerph-23-00487-s001.zip › Table S1.pdf]

Table S1: Consolidated criteria for reporting qualitative studies (COREQ): 32-item checklist

No. Item	Guide questions/description	Summary	Reported on Page #
**Domain 1: Research team and reflexivity**			
Personal Characteristics			
1. Interviewer/facilitator	Which author/s conducted the interview or focus group?	IM conducted the interviews.	6
2. Credentials	What were the researcher's credentials? E.g. PhD, MD	BMBS (Bachelor of Medicine and Bachelor of Surgery) and BMedSci	8
3. Occupation	What was their occupation at the time of the study?	Medical doctor.	8
4. Gender	Was the researcher male or female?	Female	8
5. Experience and training	What experience or training did the researcher have?	Training in qualitative research methods, interview skills, and good clinical practice (GCP).	8
Relationship with participants			
6. Relationship established	Was a relationship established prior to study commencement?	The interviewer had no prior relationship with participants.	8
7. Participant knowledge of the interviewer	What did the participants know about the researcher? e.g. personal goals, reasons for doing the research	Participants knew that the researcher was a medical doctor.	8
8. Interviewer characteristics	What characteristics were reported about the interviewer/facilitator? e.g. Bias, assumptions, reasons and interests in the research topic	Participants knew that the researcher had interests in health and medical research.	8
**Domain 2: study design**			
Theoretical framework			
9. Methodological orientation and Theory	What methodological orientation was stated to underpin the study? e.g. grounded theory, discourse analysis, ethnography, phenomenology, content analysis	Thematic analysis	7
Participant selection			
10. Sampling	How were participants selected? e.g. purposive, convenience, consecutive, snowball	Purposive sampling	6

11. Method of approach	How were participants approached? e.g. face-to-face, telephone, mail, email	Email	6
12. Sample size	How many participants were in the study?	20	6
13. Non-participation	How many people refused to participate or dropped out? Reasons?	None	6
Setting			
14. Setting of data collection	Where was the data collected? e.g. home, clinic, workplace	Interviews were online via Microsoft Teams	6
15. Presence of non-participants	Was anyone else present besides the participants and researchers?	No	6
16. Description of sample	What are the important characteristics of the sample? e.g. demographic data, date	They were health or care professionals, managers, or leaders who had accessed, used, or disseminated the digital support package.	6
Data collection			
17. Interview guide	Were questions, prompts, guides provided by the authors? Was it pilot tested?	Yes. The interview topic guide was developed by the authors, and it was pilot tested with 5 healthcare professionals who did not take part in the study.	6
18. Repeat interviews	Were repeat interviews carried out? If yes, how many?	No.	6
19. Audio/visual recording	Did the research use audio or visual recording to collect the data?	Interviews were audio recorded with consent.	6
20. Field notes	Were field notes made during and/or after the inter view or focus group?	Yes.	6
21. Duration	What was the duration of the inter views or focus group?	Average 38 minutes.	6
22. Data saturation	Was data saturation discussed?	No. We were guided by the concept of information power (Maserud et al., 2015).	6
23. Transcripts returned	Were transcripts returned to participants for comment and/or correction?	10% of transcripts returned for comment / correction (n=2)	6
**Domain 3: analysis and findings**			

Data analysis			
24. Number of data coders	How many data coders coded the data?	Two	7
25. Description of the coding tree	Did authors provide a description of the coding tree?	yes	7
26. Derivation of themes	Were themes identified in advance or derived from the data?	Derived from the data.	7
27. Software	What software, if applicable, was used to manage the data?	None.	7
28. Participant checking	Did participants provide feedback on the findings?	10% member checking and feedback was undertaken.	6
Reporting			
29. Quotations presented	Were participant quotations presented to illustrate the themes/findings? Was each quotation identified? e.g. participant number	Yes, quotations are included to illustrate each theme. All quotations are identified by number and gender.	10-18
30. Data and findings consistent	Was there consistency between the data presented and the findings?	Yes.	8-18
31. Clarity of major themes	Were major themes clearly presented in the findings?	Yes.	8-18
32. Clarity of minor themes	Is there a description of diverse cases or discussion of minor themes?	Yes.	10-18
