# Supplementary material for: Perceived Individual and Systemic Impact of a Digital Wellbeing Package for Health and Care Workers Five Years Post-Release: A Qualitative Study
Source: Int J Environ Res Public Health. 2026 Apr 13;23(4):487. doi: 10.3390/ijerph23040487 (PMC13116477; doi:10.3390/ijerph23040487)
Supplement: Supplementary file 1 [file ijerph-23-00487-s001.zip › Text S1.pdf]

Text S1. Interview topic guide.

Motivations

1. What were your reasons/goal behind wanting to access the support package? Did the E-package help fulfil those goals? How?

Experiences of the package

2. Were there any areas of the support package that you found particularly helpful?
3. Did the support package differ from your expectations (if so, how?)
4. Was there anything you wish was included, or you felt could have been done differently?

Utilisation of the package

5. Did you re-visit or save any of the content (e.g. did you bookmark certain sections to re-visit, did you make any notes/save any links/content?) Why did you save/re-visit this content?

Further research/discussions

6. Did you access any of the support links that were provided within and at the end of the package? (if so, which resources were these?)
7. Did the support package inspire you to have conversations about wellbeing? (or about any of the content covered in the support package?)

Reach

8. Did you discuss or share the support package with anyone else (e.g. colleagues, people you supervise, students, peers, managers, teams, wellbeing team, other organisations, family or friends)?
9. What were your goals when sharing the support package? Did the E-package help fulfil those goals? What content/resources/tips/techniques did you share and why?
10. Where/how did you share these (e.g. social media, word of mouth, adding it to policy documents, websites, support packages, training materials for staff)? Was it shared locally or did you also share internationally?
11. Are you aware if the E-package was incorporated within any organisation support policies or practices, at the time?

Impact

12. Did the support package bring about any changes for you personally? For example, your thoughts, mood, behaviours, or approach to self-care?
13. Did you notice any changes in your stress levels or mental wellbeing after using the support package?
14. Have you incorporated anything you learned from the support package into your work or at home? For example, have you implemented any changes into your routine or way of working?
15. Overall, what impact, if any, did this E-package have on you or others?
16. Has using the E-package had any implications for you in the short-term?
17. Has using the E-package had any implications for you in the medium-term?
18. Has using the E-package had any implications for you in the long-term?

Future use

19. Moving forward, how do you think the content from within this support package could be used to support wellbeing?
20. Do you intend on continuing to use some of the learning from the support package in the future? (If so, why and how?)
21. Did you notice any difference after accessing the E-package, in wellbeing or organisational behaviour in any individuals or teams?
