# Supplementary figures and images for: Perceived Individual and Systemic Impact of a Digital Wellbeing Package for Health and Care Workers Five Years Post-Release: A Qualitative Study
Source: Int J Environ Res Public Health. 2026 Apr 13;23(4):487. doi: 10.3390/ijerph23040487 (PMC13116477; doi:10.3390/ijerph23040487)

**Figure S2.** Integrated evidence of perceived research impact.

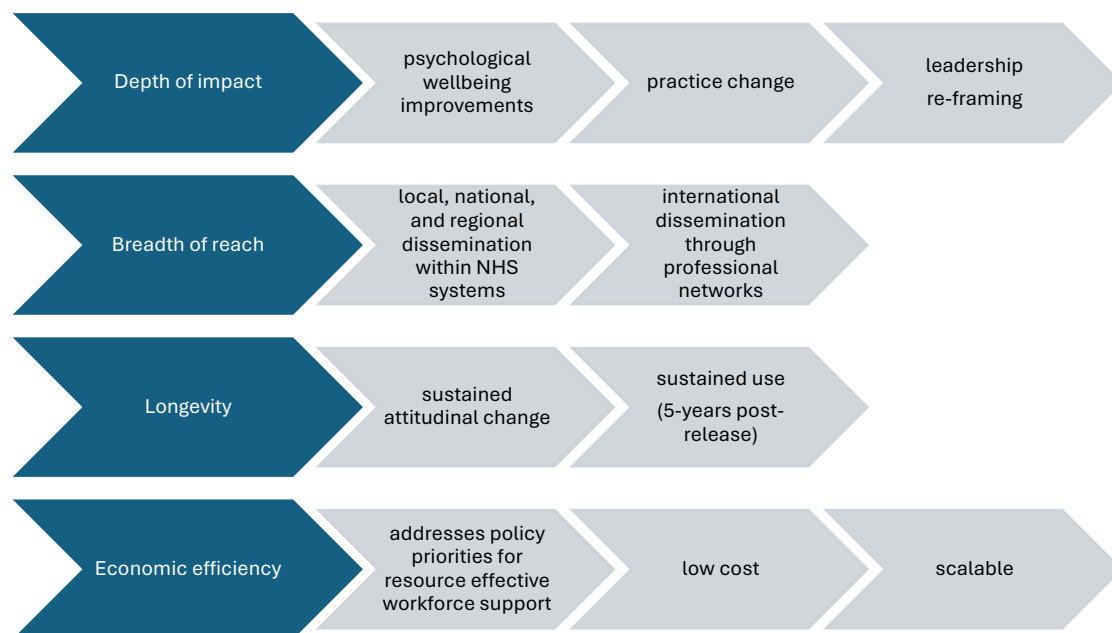

Supplement: Supplementary file 1 [file ijerph-23-00487-s001.zip › Figure S2.pdf]
